# Intracellular amyloid β oligomers impair organelle transport and induce dendritic spine loss in primary neurons

**DOI:** 10.1186/s40478-015-0230-2

**Published:** 2015-08-21

**Authors:** Tomohiro Umeda, Elisa M. Ramser, Minato Yamashita, Koichi Nakajima, Hiroshi Mori, Michael A. Silverman, Takami Tomiyama

**Affiliations:** Department of Neuroscience, Osaka City University Graduate School of Medicine, 1-4-3 Asahimachi, Abeno-ku, Osaka 545-8585 Japan; Core Research for Evolutional Science and Technology, Japan Science and Technology Agency, Kawaguchi, Japan; Department of Biological Sciences, Simon Fraser University, Burnaby, British Columbia V5A 1S6 Canada; Department of Immunology, Osaka City University Graduate School of Medicine, Osaka, Japan; Department of Clinical Neuroscience, Osaka City University Medical School, Osaka, Japan

## Abstract

**Introduction:**

Synaptic dysfunction and intracellular transport defects are early events in Alzheimer’s disease (AD). Extracellular amyloid β (Aβ) oligomers cause spine alterations and impede the transport of proteins and organelles such as brain-derived neurotrophic factor (BDNF) and mitochondria that are required for synaptic function. Meanwhile, intraneuronal accumulation of Aβ precedes its extracellular deposition and is also associated with synaptic dysfunction in AD. However, the links between intracellular Aβ, spine alteration, and mechanisms that support synaptic maintenance such as organelle trafficking are poorly understood.

**Results:**

We compared the effects of wild-type and Osaka (E693Δ)-mutant amyloid precursor proteins: the former secretes Aβ into extracellular space and the latter accumulates Aβ oligomers within cells. First we investigated the effects of intracellular Aβ oligomers on dendritic spines in primary neurons and their tau-dependency using tau knockout neurons. We found that intracellular Aβ oligomers caused a reduction in mushroom, or mature spines, independently of tau. We also found that intracellular Aβ oligomers significantly impaired the intracellular transport of BDNF, mitochondria, and recycling endosomes: cargoes essential for synaptic maintenance. A reduction in BDNF transport by intracellular Aβ oligomers was also observed in tau knockout neurons.

**Conclusions:**

Our findings indicate that intracellular Aβ oligomers likely contribute to early synaptic pathology in AD and argue against the consensus that Aβ-induced spine loss and transport defects require tau.

## Introduction

Synaptic dysfunction is an early event in Alzheimer’s disease (AD). Soluble oligomers of amyloid β (Aβ), which are generated from the amyloid precursor protein (APP), are believed to be the primary synaptotoxins in AD. According to one central view of AD pathogenesis, extracellular Aβ oligomers (eAβOs) bind plasma membrane targets to elicit pre- and postsynaptic intracellular effects (for reviews, [1, 2]). At the postsynaptic membrane, eAβOs interact with glutamate receptors and dysregulate calcium influx to impair long-term potentiation (LTP) and enhance long-term depression (LTD) [3–6]. eAβO binding also alters spine morphology and decreases spine density [7, 8]. In axons, eAβOs impair transport of cargoes such as mitochondria and vesicles containing brain-derived neurotrophic factor (BDNF) [9–11], which are both required for neuronal form and function. Mitochondria are needed at presynaptic boutons to maintain neurotransmission by producing ATP and buffering synaptic calcium (Ca^2+^) [12–14]. Once secreted from axon terminals, BDNF increases spine density and the proportion of mature spines by interacting with postsynaptic TrkB receptors at the target cell membrane [15, 16]. Thus, impaired transport of mitochondria and BDNF might contribute to synaptic dysfunction in AD [17, 18].

Although a role for eAβOs in causing AD-like toxicity is well established, several studies have revealed that intraneuronal accumulation of Aβ is also toxic and precedes its extracellular deposition in patients and model mice of AD [19–24]. In model mice that overexpress mutant human APP, synaptic dysfunction [22, 25], spine morphology alteration [26], and axonal transport defects [27] were observed in association with intracellular Aβ oligomers (iAβOs). These pathological changes, however, may have been induced by overexpression of human APP or undetected extracellular Aβ. Cellular mechanisms that underlie iAβO-induced synaptic dysfunction remain uncharacterized. Furthermore, although it is widely reported that tau is required for eAβO toxicity (for a review, [28]), whether iAβO toxicity is tau-dependent has not yet been investigated.

A valuable model for studying iAβOs is an APP mutation identified in familial AD. The Osaka (E693Δ) mutation in APP induces iAβO accumulation without detection of Aβ fibrillization *in vitro* and without detectable Aβ plaque formation in AD patients or mouse models [29–31]. Intracellularly, the Osaka mutation-induced iAβOs lead to endoplasmic reticulum stress and damage of mitochondria and organelles within the endosomal/lysosomal system [32].

Here, we determined and compared the effects of wild-type APP (APP_WT_) and Osaka-mutant APP (APP_OSK_) on dendritic spine morphology and intracellular transport of organelles required for synaptic maintenance and function. We found that iAβOs reduced the number of mature spines and impaired transport of BDNF, mitochondria, and recycling endosomes in hippocampal neurons expressing APP_OSK_. Notably, spine reduction and impairment of BDNF transport occurs independently of tau. These results advance our understanding of early AD synaptic pathology because iAβO accumulation precedes extracellular amyloid deposition in patients and AD model mice. Our findings may promote development of effective therapeutic compounds for AD prevention and treatment.

## Materials and methods

### Preparation of primary neurons

Mouse primary neurons were prepared from embryos of wild-type (*MAPT* +/+) and tau knockout (*MAPT* −/−) mice (Jackson Laboratory, Bar Harbor, ME) at embryonic day 18 (E18). Hippocampal tissues were dissected in ice-cold Hank’s balanced salt solution (HBSS; Sigma-Aldrich, St. Louis, MO) and incubated in 1 ml of papain solution (2 mg/ml in HBSS) at 37 °C for 30 min with gentle mixing. After being washed with 5 ml of 50 % horse serum in HBSS once, 5 ml of HBSS twice, and 4 ml of neuronal culture medium (Neurobasal, Electro medium supplemented with B27, Electro and 500 μM L-glutamine; all from GIBCO, Life Technologies, Carlsbad, CA) once, the tissues were dissociated into cells by pipetting several times with a Pasteur pipet in 2 ml of neuronal culture medium. The cell suspensions were plated onto poly-L-lysine-coated coverslips in 6-well culture plates at a density of 170,000 cells/2 ml/well. For transport analyses, primary neurons from wild-type and tau knockout mice, and wild-type rats were prepared from embryos at E16 and E18, respectively, as described [33]. Neurons were cultured on coverslips in Neurobasal medium supplemented with B27 at a density of 250,000 cells/5 ml in 6 cm dishes. The astrocyte feeder layer for the neuronal co-culture was generated using neural progenitor cells as described previously [34].

### Expression vectors

The pCI-APP construct was prepared using a pCI vector (Promega, Madison, WI) as described previously [29]. A pEGFP (enhanced green fluorescent protein)-N2 vector was obtained from Clontech (Takara Bio Inc. Otsu, Japan). The pIRES2-APP-EGFP vectors were made by amplifying APP_WT_ and APP_OSK_ using a forward primer containing an Nhe I restriction site (underlined) 5′-AATTAATTAA**GCTAGC**GCCACCATGGGGGCTGCCCGGTTTGGCACTGCT-3′ and a reverse primer containing a Sac II restriction site 5′- TTAATTAATT**CCGCGG**CTAGTTCTGCATCTGCTCAAAGAACTTGTAGGTTGG-3′ for subcloning into pIRES2-EGFP (Takara Clontech Bio Inc. Otsu, Japan). Plasmid composition was confirmed by sequencing. The pβ-actin-BDNF-mRFP (monomeric red fluorescent protein) and pβ-actin-eBFP (enhanced blue fluorescent protein) vectors were a kind gift from Dr. G. Banker (Oregon Health and Sciences University). The pcDNA3 Mt-eYFP (enhanced yellow fluorescent protein) construct was designed to express the COX IV mitochondrial-targeting sequence-EYFP fusion proteins and kindly gifted from Dr. G. Rintoul (Simon Fraser University). The JPA5-TfR -GFP vector is described in Burack et al. [35].

### Analyses of APP expression

Mouse primary neurons were cultured for 21 days in vitro. The cells were cotransfected with pCI-APP (APP_WT_, APP_OSK_, or empty) and pEGFP-N2 using a Lipofectamine2000 reagent (Invitrogen, Life Technologies). Transfection was performed in the presence of 0.5 μM kynurenic acid (Sigma-Aldrich) to lessen excitotoxic cell damage. Cells expressed the transgenes for 2 days. For immunocytochemical analysis of APP expression, the cells were fixed with 4 % paraformaldehyde in PBS at room temperature for 30 min and permeabilized by immersion in 0.05 % Tween-20 in PBS for a moment. After a brief wash, the cells were blocked with 20 % calf serum in PBS at room temperature for 1 h. The cells were then stained with human APP-specific antibody 6E10 (Covance, Berkeley, CA) or Aβ oligomer–specific antibody 11A1 (IBL, Fujioka, Japan) at room temperature for 1 h followed by Rhodamine-conjugated anti-mouse IgG antibody (Jackson ImmunoResearch Labs, West Grove, PA) at room temperature for 20 min. The stained specimens were mounted with VECTASHIELD mounting medium with DAPI (H-1500; Vector Laboratories, Burlingame, CA) and viewed under a Leica TCS SP5 confocal laser microscope (Leica, Wetzlar, germany). For Western blot analysis of APP expression, the cells were lysed in 1 % Triton X-100/Tris-buffered saline (100 mM Tris–HCl, pH 7.6, 150 mM NaCl) containing protease inhibitor cocktail P8340 (Sigma-Aldrich). The lysates were separated by SDS-PAGE and transferred onto polyvinylidene difluoride membranes. Human APP and actin were stained with 6E10 and rabbit anti-actin antibody (Sigma-Aldrich) followed by horseradish peroxidase–conjugated secondary antibodies and chemiluminescent peroxidase substrate (Millipore, Billerica, MA). Signals were visualized and quantified using a LAS-3000 luminescent image analyzer (Fujifilm, Tokyo, Japan).

### Analyses of dendritic spines

Mouse primary neurons transfected with pCI-APP and pEGFP-N2 were fixed with 4 % paraformaldehyde after a 2-day culture. The fixed cells were mounted, and the images were taken using a Leica TCS SP5 confocal laser microscope. Dendritic spines were classified into four groups by the criteria as follows: mushroom, the length ≤ 5 μm and the ratio of neck width/head width ≥ 1.5; stubby, the length ≤ 1 μm and the ratio of neck width/head width < 1.5; thin, 1 < the length ≤ 5 μm and the ratio of neck width/head width < 1.5; and fillopodia, the length ≥ 1.5 μm without a head [36, 37]. Three to seven independent cultures were made for each APP_WT_-, APP_OSK_-, and mock transfection. One transfected cell was chosen from each culture and analyzed for spines with 3 to 12 dendrites per cell.

To study the effects of extracellular Aβ on spines, we first determined the levels of Aβ secreted from cells into culture media. Culture media of mouse primary neurons transfected with pCI-APP were harvested 2 days after transfection. Aβ concentrations in the media were measured using a human/mouse Aβ40 ELISA kit (Wako Pure Chemical Industries, Osaka, Japan). Then, mouse primary neurons transfected with pEGFP-N2 alone (without pCI-APP) were cultured for 2 days in the presence of synthetic wild-type Aβ42 or Osaka (E22Δ)-mutant Aβ42 (41 amino acids) peptide (both from Peptide Institute, Mino, Japan) at various concentrations. After cell fixation, dendritic spines were analyzed as described above.

### Analyses of axonal and dendritic transport

Mouse and rat primary neurons were cultured for 10–12 days *in vitro*. For analysis of BDNF transport, mouse primary neurons were doubly transfected with pIRES2-APP-EGFP and pβ-actin-BDNF-mRFP. For analysis of mitochondria transport, rat primary neurons were triply transfected with pCI-APP, pcDNA3 Mt-eYFP, and pmUBa-eBFP. To analyze recycling endosome transport, we chose the transferrin receptor (TfR) as a marker for recycling endosomes and used pCI-APP, JPA5-TfR-GFP, and pmUBa-eBFP in transfection of rat primary neurons. The soluble GFP and BFP are distributed throughout the cell body and processes enabling us to determine the orientation of the cell body relative to the axon, thus, distinguishing anterograde and retrograde transport. All transfections were done in the presence of 0.5 μM kynurenic acid as described above. Two days after transfection, BDNF/mitochondria/TfR transport was analyzed using a standard wide-field fluorescence microscope equipped with a cooled charge-coupled device camera and controlled by MetaMorph (Molecular Devices, Sunnyvale, CA) according to Kwinter et al. [38]. BDNF imaging was recorded by the “stream acquisition module”, and mitochondria and TfR imaging were recorded by the “acquire timelapse module” in MetaMorph. Briefly, cells were sealed in a heated imaging chamber, and streaming recordings of BDNF were acquired from triple transfectants for 25 s (250-msec exposures). Frames were captured continuously for 300 s (400-msec exposures) for mitochondria transport and 100 s (500-msec exposures) for TfR transport. This captured dozens of transport events per cell in 100-μm segments of the axon and 45-μm segments of the dendrite. Axons and dendrites were initially identified based on morphology and confirmed retrospectively by immunostaining MAP2, a dendrite-specific microtubule-associated protein, with mouse anti-MAP2 antibody (Millipore). Vesicle flux was obtained through tracing kymographs in MetaMorph. Vesicle flux was defined as the total distance traveled by vesicles standardized by the length and duration of each movie (in micron-minutes): ∑_*i* = 1_^*n*^*d*_*i*_/(*ℓ* × *t*), where *d* is the individual vesicle run lengths, *ℓ* is the length of axon or dendrite observed, and *t* is the duration of the observation [38].

### Statistical analyses

All values obtained are expressed as the mean ± SEM. Comparisons of means among multiple groups were performed using Fisher’s PLSD test following ANOVA. Differences with a *p* value of <0.05 were considered significant.

## Results

### Dendritic spines are altered in neurons accumulating iAβOs

**Fig. 1:**
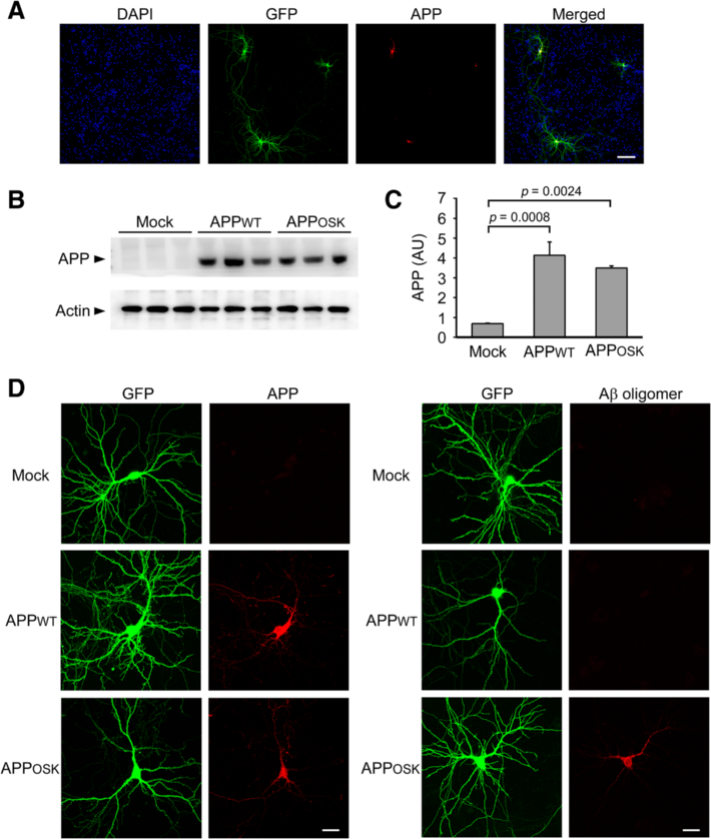
APP expression and Aβ oligomer accumulation in APP-transfected neurons. Mouse primary neurons were transfected with two plasmid vectors to simultaneously express APP and GFP. **a** Cells were stained with DAPI and human APP-specific 6E10 antibody (red). All GFP-positive neurons expressed human APP. Scale bar, 200 μm. **b** Cell homogenates were subjected to Western blot with 6E10 and anti-actin antibodies. **c** No significant difference in the levels of human APP expression between APP_WT_- and APP_OSK_-transfectants. AU, arbitrary unit. **d** Cells were stained with 6E10 (red) or Aβ oligomer-specific 11A1 antibody (red). Only APP_OSK_-expressing neurons accumulated abundant iAβOs. Scale bar, 30 μm

**Fig. 2:**
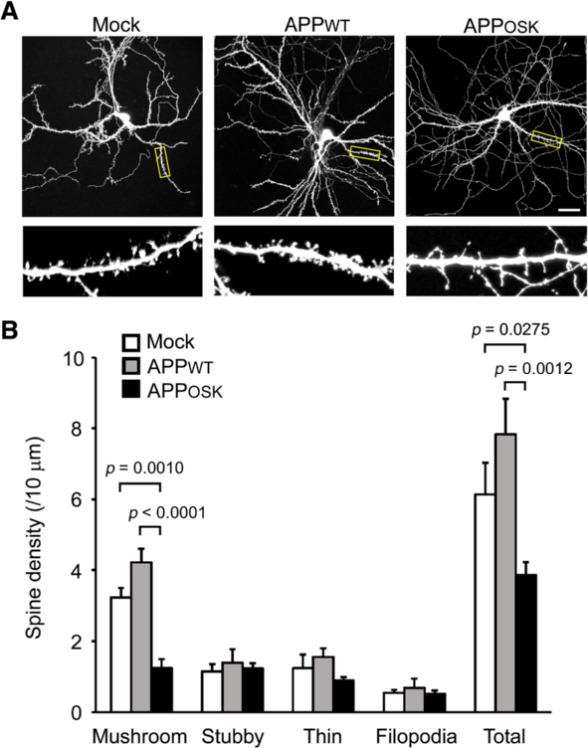
Spine alteration in APP_OSK_-expressing neurons. **a** Mouse primary neurons were doubly transfected with APP and GFP. Scale bar, 30 μm. Lower panels, enlarged views of the dendrites surrounded with a square. **b** Individual spines on dendrites of GFP-positive neurons were classified into the four types: mushroom, stubby, thin, and filopodia-like protrusions. Compared with mock-transfectants, APP_WT_-expressing cells showed a small increase the number of total and mushroom-type spines with no significant changes in other types. In contrast, APP_OSK_-expressing cells exhibited a significant reduction in the number of total and mushroom-type spines, but no significant changes in other types

Spines are generally classified into four types based on the shape: stubby (type-I), mushroom (type-II), thin (type-III), and filopodia-like protrusions. Mushroom spines are stable and represent mature synapses, while thin spines are transient and maintain structural plasticity [39]. Stubby spines are thought to be immature, and filopodia-like protrusions lack synapses [39]. Due to the reduction in synapses found in AD brains, we asked if there is a change in the density and morphology of dendritic spines in APP expressing neurons. Mouse primary neurons were transfected with two plasmid vectors to simultaneously express APP and GFP, the latter was introduced to visualize spines. By staining cells with human APP-specific 6E10 antibody, we confirmed that all GFP-positive neurons expressed human APP (Fig. 1a). Western blot analysis with the 6E10 antibody indicated no significant difference in the levels of human APP expression between APP_WT_- and APP_OSK_-transfectants (Fig. 1b and c). Importantly, only APP_OSK_-expressing neurons accumulated abundant iAβOs, which were visualized by staining with the Aβ oligomer-specific 11A1 antibody (Fig. 1d). We classified individual spines on dendrites of GFP-positive neurons into the four types, and compared the numbers of total and each type spines among APP_OSK_-expressing, APP_WT_-expressing, and mock-transfected neurons (Fig. 2). Compared with mock-transfectants, APP_WT_-expressing cells showed a small increase in the number of total and mushroom-type spines with no significant changes in other types. In contrast, APP_OSK_-expressing cells exhibited a significant reduction in the number of total and mushroom-type spines, but no significant changes in other types. Taken together, these results suggest that the accumulation of iAβOs markedly decreases spine density, particularly the mature mushroom-type spines.

### Spine reduction is not a result of extracellular Aβ

Altered spine density and morphology may have been induced by extracellular Aβ secreted from cells. To test this possibility, we first measured the levels of Aβ in the culture media using a human/mouse Aβ40 ELISA kit. The culture media of mock-, APP_WT_-, and APP_OSK_-transfectants contained about 89.5 ± 17.1, 183.5 ± 15.1, and 63.8 ± 4.5 pM of Aβ40, respectively. This is consistent with our previous finding that the Osaka mutation reduced Aβ secretion [30].

**Fig. 3:**
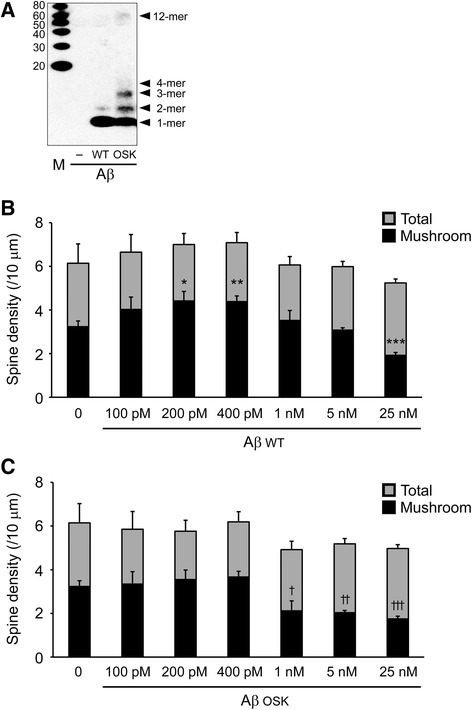
Effects of extracellular Aβ on spines. Synthetic wild-type (WT) or Osaka-mutant (OSK) Aβ42 peptides were added into culture media of GFP-expressing neurons at various concentrations. **a** Before adding the peptides, Aβ oligomer formation in the peptide solutions were examined by Western blot with 6E10 antibody. Aβ WT peptide showed mainly monomers and faintly dimers, whereas Aβ OSK peptide exhibited abundant monomers as well as dimers and trimers and faintly tetramers and 12-mers. **b**, **c** After a 2-day culture in the presence of Aβ peptides, spine densities and types were measured. Aβ WT peptide (**b**) increased total and mushroom-type spines at physiological, low concentrations from 100 pM to 400 pM. In contrast, Aβ OSK peptide (**c**) did not affect spine morphology at those concentrations. However, both Aβ WT and Aβ OSK peptides significantly decreased total and mushroom-type spines at higher concentrations: at 25 nM of Aβ WT peptide and over 1 nM of Aβ OSK peptide

Subsequently we added synthetic Aβ42 peptide into culture media of GFP-expressing neurons at various concentrations. Before adding the peptides, we examined Aβ oligomer formation in the peptide solutions by Western blot using the 6E10 antibody. Wild-type Aβ42 peptide showed mainly monomers and with a faint signal for dimers, whereas the Osaka-mutant Aβ42 peptide exhibited abundant monomers as well as dimers, trimers, tetramers, and 12-mers (Fig. 3a). After 48 h exposure to Aβ peptides, spine densities and types were measured in primary neurons. Unexpectedly, wild-type Aβ42 peptide increased total and mushroom-type spines at physiological, low concentrations (100 pM to 400 pM; Fig. 3b) [40, 41]. However, such a trophic effect was attenuated at concentrations over 1 nM. At 25 nM, wild-type peptide significantly decreased total and mushroom-type spines. In contrast, the Osaka-mutant Aβ42 peptide did not affect spine morphology at low concentrations (100 pM – 400 pM), yet significantly decreased total and mushroom-type spines at higher concentrations over 1 nM (Fig. 3c). These results are consistent with our previous observation that wild-type, but not Osaka-mutant, Aβ42 peptide showed a trophic effect on synaptophysin levels in cultured mouse hippocampal slices [42]. The present result with wild-type Aβ peptide implies that the increase in total and mushroom-type spines observed in APP_WT_-expressing neurons (Fig. 2) might be induced by extracellular Aβ secreted from the cells. On the contrary, the result with the Osaka-mutant Aβ peptide suggests that the spine alterations observed in APP_OSK_-expressing neurons (Fig. 2) could be attributed to iAβOs.

### iAβO-induced spine alteration is independent of tau

**Fig. 4:**
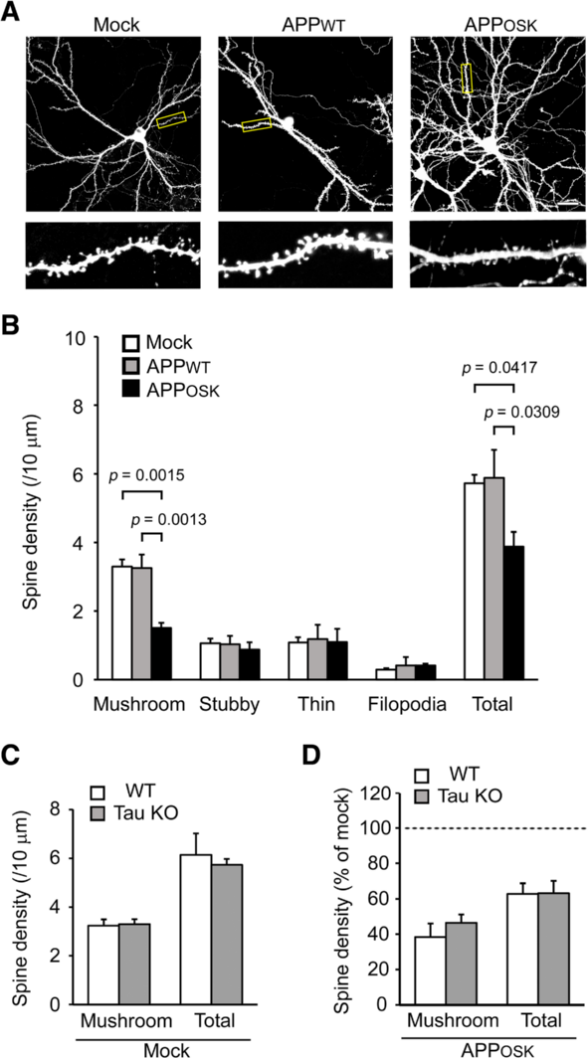
Spine alteration in APP_OSK_-expressing tau knockout neurons. **a** Primary neurons were prepared from tau knockout mice and doubly transfected with APP and GFP. Scale bar, 30 μm. Lower panels, enlarged views of the dendrites surrounded with a square. **b** Spine density and morphology in GFP-positive neurons were analyzed. APP_WT_-expressing cells showed no differences in the number of total and each type spine compared to mock-transfectants. In contrast, APP_OSK_-expressing cells exhibited a significant reduction in the number of total and mushroom-type spines with no significant changes in other types. **c**, **d** Comparison of spine alteration between wild-type (Fig. 2) and tau knockout neurons expressing APP_OSK_. **c** There were no differences in the density of mushroom-type and total spines between wild-type and tau knockout neurons. **d** APP_OSK_ expression reduced the spine density in both wild-type and tau knockout neurons: No differences in the reduction of mushroom-type (*p* = 0.4835) and total spines (*p* = 0.9702) between these neurons

Whether extracellular Aβ requires tau to mediate changes in postsynaptic structure remain a topic of debate. One model suggests that postsynaptic defects occur upstream of significant changes in tau phosphorylation [43], yet recent studies indicate tau hyperphosphorylation and mislocalization leads to postsynaptic deficits [44–46]. However, it is unknown if tau is required for iAβOs to exhibit toxic effect on spines. Primary neurons were prepared from tau knockout mice and doubly transfected with APP and GFP. We confirmed again that all GFP-positive neurons expressed human APP and that only APP_OSK_-expressing neurons accumulated abundant iAβOs (data not shown). Similar to the experiments in wild-type neurons we analyzed spine density and morphology in GFP-positive neurons. APP_WT_-expressing cells showed no differences in the number of total spines and morphologically distinct spines compared to mock-transfectants (Fig. 4a, b). The trophic effect of APP_WT_ expression on total and mushroom-type spines was not seen in tau knockout neurons. In contrast, APP_OSK_-expressing cells exhibited a significant reduction in the number of total and mushroom-type spines with no significant changes in other types. Finally, we observed no significant differences in spine alteration between wild-type and tau knockout neurons expressing APP_OSK_ (Fig. 4c, d). These results indicate that iAβO-induced spine alteration is independent of tau.

### iAβOs disrupt axonal and dendritic transport of BDNF

What is the cellular mechanism underlying the spine alterations caused by iAβOs? Notably, BDNF is known to increase spine density and the proportion of mature spines. Furthermore, reduced levels of BDNF correlate with AD progression [17, 47]. We speculated that iAβOs disrupt vesicular transport of BDNF to sites of release in axons and dendrites, thereby inducing spine alteration.

**Fig. 5:**
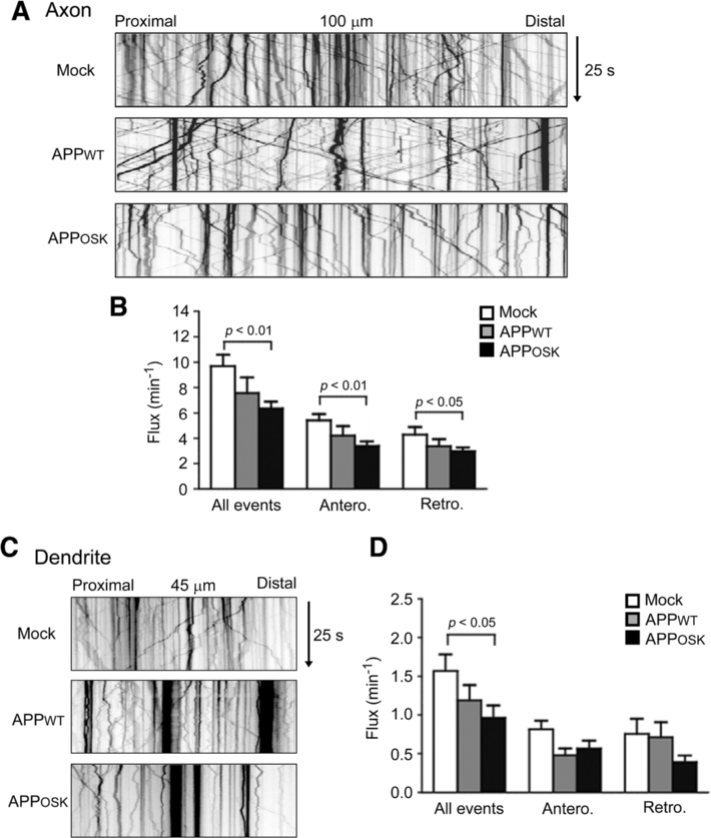
Axonal and dendritic transport of BDNF is impaired in APP_OSK_-expressing neurons. Wild-type mouse primary neurons were doubly transfected with APP-EGFP and BDNF-mRFP. Transport of BDNF in living neurons was recorded for 25 s, and those in 100-μm segments of the axon (**a**) and 45-μm segments of the dendrite (**c**) were analyzed. Axons and dendrites were initially identified based on morphology and confirmed retrospectively by immunostaining MAP2. **b** Compared with mock-transfectants, both APP_WT_- and APP_OSK_-expressing cells showed a decrease of bidirectional transport of BDNF in axons, but the differences were significant only in APP_OSK_-expressing cells. **d** Similarly, a decrease of bidirectional transport of BDNF in dendrites was observed in both APP_WT_- and APP_OSK_-expressing cells, but significant reduction was observed only for total event in APP_OSK_-expressing cells

To test this hypothesis, we analyzed axonal and dendritic transport of BDNF in living neurons. Wild-type mouse primary neurons were transfected with APP-EGFP and BDNF-mRFP. After video recording, neurons were fixed and stained with an antibody to MAP2, a dendrite-specific microtubule-associated protein, to distinguish dendrites from axons. Compared with mock-transfectants, both APP_WT_- and APP_OSK_-expressing cells showed a decrease of anterograde, retrograde, and total axonal flux of BDNF, but the differences were significant only in APP_OSK_-expressing cells (Fig. 5a, b; flux is an index of transport described in the Materials and Methods). Similar results were obtained in dendrites: A decrease in bidirectional transport of BDNF was observed in both APP_WT_- and APP_OSK_-expressing neurons, but significant reduction was observed only for total transport in APP_OSK_-expressing neurons (Fig. 5c, d). These results support our speculation that iAβOs cause spine alterations via the blockade of BDNF transport that is required to supply sites of release.

### iAβOs disrupt axonal and dendritic transport of BDNF independent of tau

**Fig. 6:**
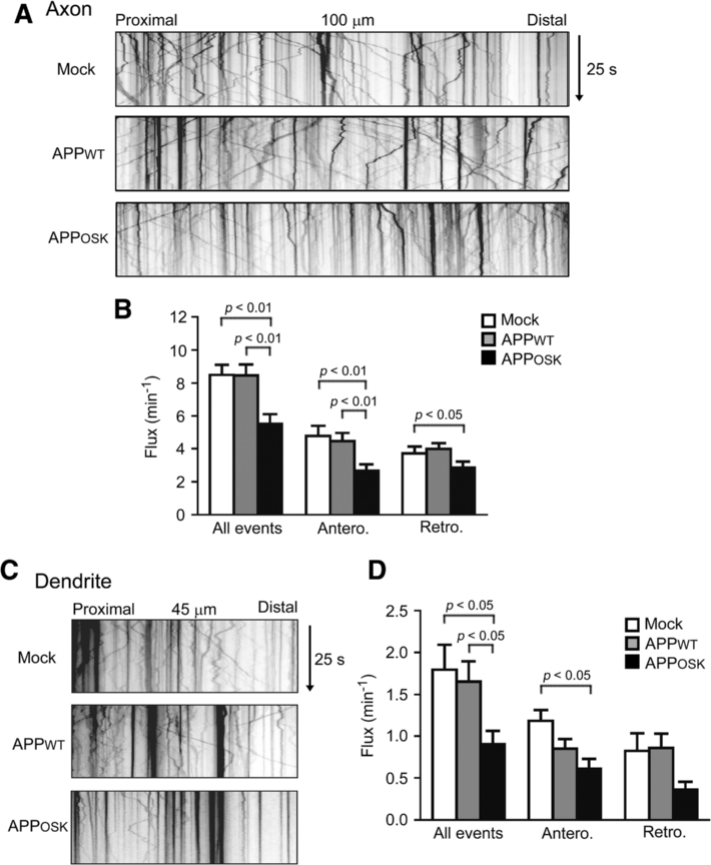
Axonal and dendritic transport of BDNF is impaired in APP_OSK_-expressing tau knockout neurons. Tau knockout mouse primary neurons were doubly transfected with APP-EGFP and BDNF-mRFP. Transport of BDNF in living neurons was recorded for 25 s, and those in 100-μm segments of the axon (**a**) and 45-μm segments of the dendrite (**c**) were analyzed. **b** Compared with mock-transfectants, APP_WT_-expressing cells showed no differences in BDNF transport in axons. In contrast, APP_OSK_-expressing cells showed a significant decrease of anterograde, retrograde, and total axonal flux of BDNF. **d** In dendrites, both APP_WT_- and APP_OSK_-expressing cells showed a decrease of BDNF transport, but the differences were significant only in APP_OSK_-expressing cells

We showed that iAβO-induced spine alteration is independent of tau (Fig 4), but it is unknown if this tau-independence extends to other cellular mechanisms such as intracellular transport. To determine if iAβO-induced BDNF transport defects are tau independent, we analyzed transport of BDNF in tau knockout mouse neurons. APP_WT_-expressing cells showed no differences in BDNF transport in axons compared to mock-transfectants (Fig. 6a, b). In contrast, APP_OSK_-expressing cells showed a significant decrease of anterograde, retrograde, and total axonal flux of BDNF, similar to wild-type neurons (Fig. 5). In the dendrites of APP_OSK_-expressing neurons, BDNF transport was also significantly reduced (Fig. 6c, d). Similar to the effects of eAβOs [48, 49], these results indicate that iAβOs impair BDNF transport independent of tau.

### Axonal transport and dendritic distribution of mitochondria are disrupted by iAβOs

**Fig. 7:**
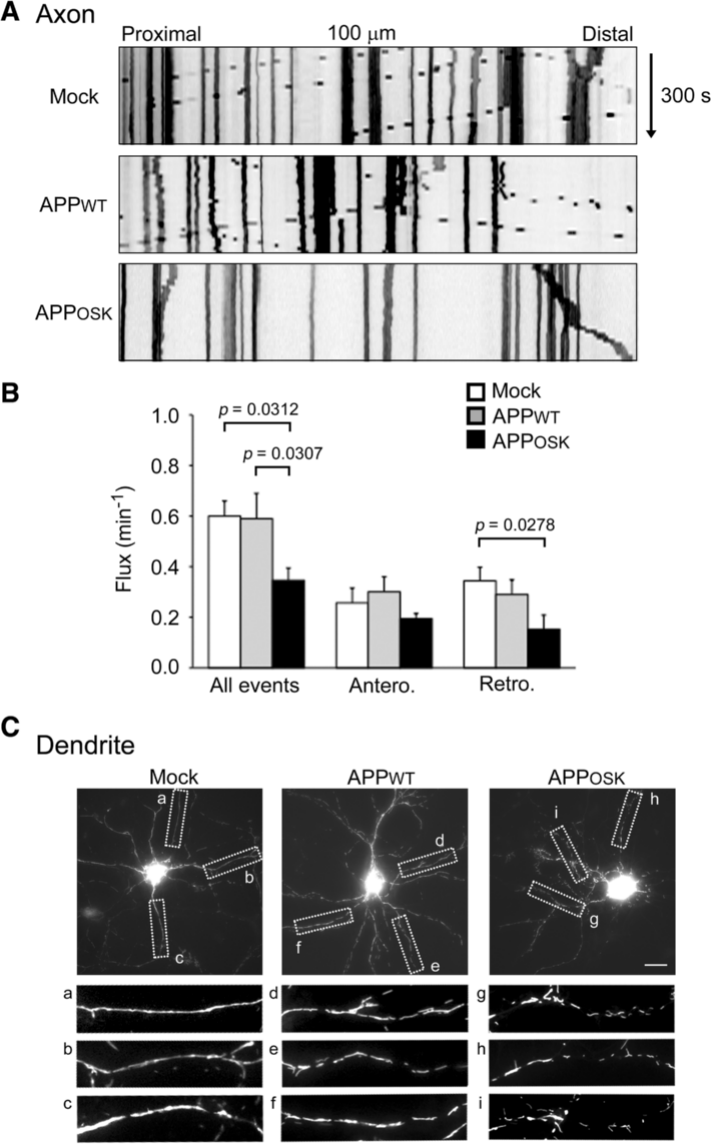
Impaired axonal transport and aberrant dendritic distribution of mitochondria in APP_OSK_-expressing neurons. Rat primary neurons were triply transfected with APP, Mt-eYFP, and BFP. **a** Transport of mitochondria in living neurons was recorded for 300 s, and those in 100-μm segments of the axon were analyzed. **b** Compared with mock-transfectants, bidirectional transport of mitochondria in axons was reduced in APP_OSK_-, but not APP_WT_-, expressing cells. **c** In dendrites, mitochondria were evenly distributed along the dendritic shafts in mock-transfectants (a-c) and APP_WT_-expressing cells (d-f). In contrast, mitochondria in dendrites of APP_OSK_-expressing cells (g-i) were of shorter length and unevenly distributed. Scale bar, 20 μm

Mitochondria are essential for synaptic function as they provide ATP for synaptic transmission and contribute to local calcium buffering [13]. Because mitochondrial and BDNF transport are both microtubule-dependent, it is likely that mitochondrial transport is also affected by iAβOs. To test this possibility, rat primary neurons were triply transfected with APP, Mt-eYFP, and BFP. Live imaging analysis revealed that bidirectional transport of mitochondria in axons was reduced in APP_OSK_-, but not APP_WT_-, expressing cells compared with mock-transfectants (Fig. 7a, b). In dendrites, mitochondria were evenly distributed along the dendritic shafts in mock-transfectants and APP_WT_-expressing cells (Fig. 7c). In contrast, mitochondria in dendrites of APP_OSK_-expressing cells were of shorter length and unevenly distributed, implying aberrant transport or a change in fission-fusion dynamics [50]. The latter finding may support our previous observation that mitochondrial network was fragmented in COS-7 cells expressing APP_OSK_ [32]. These results indicate that the disruption of mitochondria transport may also contribute to the spine alterations observed in neurons accumulating iAβOs.

### Dendritic transport of TfR-labeled endosomes is impaired by iAβOs

**Fig. 8:**
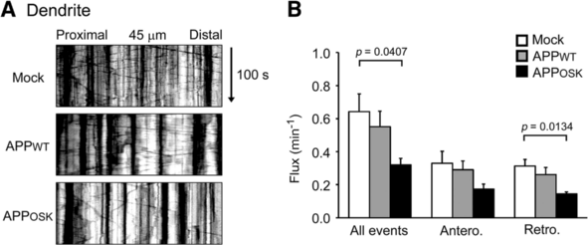
Impaired dendritic transport of recycling endosomes in APP_OSK_-expressing neurons. Rat primary neurons were triply transfected with APP, TfR-GFP, and BFP. **a** Transports of TfR in living neurons were recorded for 100 s, and those in 45-μm segments of the dendrite were analyzed. **b** Compared with mock-transfectants, bidirectional transport of TfR-positive vesicles in dendrites was reduced in APP_OSK_-, but not APP_WT_-, expressing cells

Spine formation and growth requires membrane trafficking mediated by recycling endosomes [51]. Thus, we examined the effects of iAβOs on the dendritic transport of recycling endosomes. Rat primary neurons were triply transfected with APP, TfR-GFP, and BFP. TfR is a marker protein localized to recycling endosomes. Live imaging analysis revealed that bidirectional transport of TfR-positive vesicles in dendrites was reduced in APP_OSK_-, but not APP_WT_-, expressing cells compared with mock-transfectants (Fig. 8). These results suggest that the impairment of recycling endosome transport may also contribute to the spine alteration caused by iAβOs.

## Discussion

In the present study we showed that iAβOs, generated by APP_OSK_ expression in cultured neurons, impaired axonal and dendritic transport of BDNF, mitochondria, and dendritic recycling endosomes (Figs. 5, 7 and 8). Because these cargoes are critical for spine formation and maintenance, reduced trafficking may have led to the observed decreases in spine density and in the number of mature mushroom spines (Fig. 2). Our results are significant because intraneuronal accumulation of Aβ precedes its extracellular deposition in patients and AD model mice; thus, iAβOs likely contribute to the early synaptic pathology in AD. Moreover, multiple lines of evidence demonstrate that trafficking defects are either an early cellular pathology or even causal in AD [52], underscoring the importance of defining iAβO mechanisms of action.

BDNF transport defects in APP_OSK_ neurons may reduce the amount of BDNF available for secretion, and in turn, compromise dendritic spine maturation and density. Spines are the primary site of excitatory input on neurons, and a reduced spine number and changes in morphology contribute to synaptic pathology in AD. BDNF secreted from cells binds to and activates TrkB receptors that are located on both presynaptic axon terminals and postsynaptic dendritic spines of glutamatergic synapses [53]. BDNF-induced TrkB signaling modulates synaptic transmission by enhancing presynaptic glutamate release and increasing the open probability of postsynaptic NMDA receptor ion channel [53]. Increased NMDA receptor currents activate the Rac1 (Ras-related C3 botulinum toxin substrate 1) pathway and suppress cofilin, an actin-depolymerizing factor, thereby promoting spine growth and stabilization [54]. Thus, iAβO-induced transport impairment may reduce the amount of BDNF available for secretion, leading to synaptic impairment and spine reduction.

We also found that iAβOs impair transport of mitochondria and recycling endosomes, which are essential for spine development and maintenance. Mitochondria translocate to pre- and postsynaptic regions to supply ATP for neurotransmission [13]. A reduction in ATP availability may reduce presynaptic secretion of essential signaling molecules such as glutamate and BDNF, and thereby, as similarly described above, impair postsynaptic signaling of cascades such as Ca^2+^/calmodulin-dependent protein kinase II (CaMKII) and Rac 1 that are required for spine maintenance [54–56]. A role for dendritic mitochondria is to buffer synaptic calcium; excess calcium may negatively regulate actin-binding proteins that are required for maintaining for spine density and plasticity (discussed below) [12, 57]. Thus, perturbations in mitochondrial motility or morphology induced by iAβOs likely diminish spine structure stemming from multiple mechanisms. Recycling endosomes also play an essential role in spine morphology. Elegant studies from the Ehlers laboratory demonstrated that active transport of recycling endosomes supplies necessary plasma membrane lipids and proteins required to support spine structure and function [51]. Taken together, iAβO-induced intracellular transport disruption is likely an important contributor to mature spine loss in APP_OSK_-expressing neurons.

iAβOs may disrupt intracellular transport by perturbing motor function through second message cascades or by directly binding to motor proteins. eAβO binding to membrane targets results in changes in kinase and phosphatase activity leading to transport blockade of organelles, including mitochondria and BDNF-containing vesicles [9–11, 48, 58–60]. Similarly, organelle transport is reduced in squid axoplasm perfused with AβOs via activation of casein kinase 2 [61], providing evidence that iAβOs impinge on signaling cascades that reduce trafficking. It is likely that APP_OSK_ dysregulates intracellular Ca^2+^ signaling via ER stress and mitochondrial damage [32] that may ultimately have negative consequences on the mechanisms that regulate transport. For example, mitochondria transport is governed by Miro-Milton-Kinesin-I in a Ca^2+^-dependent mechanism, where Ca^2+^ binding to Miro inhibits Kinesin-I-based motility [62]. It is thus possible that the APP_OSK_ effects on intracellular Ca^2+^ result in a blockade of mitochondria transport. Moreover, Ca^2+^ signaling may represent a general mechanism for the regulation of microtubule-based transport. The transport of dense core vesicles containing neuropeptides [63] and the dendritic kinesin, KIF17, ferrying the NR2B glutamate receptor subunit [64], are also subject to Ca^2+^ regulation, demonstrating that perturbations in Ca^2+^ homeostasis likely have broader effects on organelle trafficking. A second possible mechanism of transport disruption is the direct binding of iAβOs or Aβ to motor proteins [65]. Ari et al. demonstrate mislocalization of NMDA receptors and NGF/NTR (p75) at the post-synaptic membrane due to intracellular Aβ binding the mitotic kinesin Eg 5 [66].

Despite a plethora of recent reports, the role of tau in transport disruption is still a matter of debate. In vivo studies demonstrate that axonal transport is unaffected by tau overexpression or suppression, or by moderate amounts of hyperphosphorylated tau [67, 68]. In primary culture, we have demonstrated that eAβO-induced BDNF-transport disruption is independent of tau [48, 49]. However, other studies suggest that phospho-tau inhibits fast axonal transport (FAT) by interacting directly with motor-cargo complexes or initiating aberrant signaling cascades that alter FAT dynamics [69, 70], and that tau reduction prevents AβO-induced defects in mitochondria and neurotrophin receptor TrkA transport [10, 60]. It is possible that transport defects are motor and/or cargo dependent. For example, mitochondria are transported primarily by KIF5 whereas BDNF is transported primarily by KIF1A [71]. Kinesins may be differentially affected by hyperphosphorylated tau, thus, we cannot rule out that in our studies the reduction in mitochondria and endosome transport is tau-dependent. How APP_OSK_ and tau influences organelle transport is a focus of ongoing studies.

Similar to the initiation of transport deficits, iAβO-induced signaling cascades may compromise dendritic spine maturation and density. Actin, a critical structural component of spines, is tightly regulated by actin-binding proteins and their associated kinases and phosphatases [72]. eAβOs reduce spine density and alter morphology by at least two mechanisms: one is the mislocalization of p21-activated kinase (PAK), and another is the activation of the calcineurin, a calcium-dependent phosphatase implicated in AD. Changes in these signaling cascades alter the dynamics of actin-binding proteins needed to maintain and stabilize spine actin [73–75]. Through a yet unexplored mechanism, iAβOs may also impinge on the regulation of actin-binding proteins that lead to spine retraction. Consistent with our previous findings for eAβOs, we show that iAβO-induced BDNF transport defects and spine loss occur independent of tau. These effects are likely caused by signaling cascade activation, such as the calcineurin-GSK3β pathway, that persists in the absence of tau [48]. Because tau knockout mice do not exhibit any serious health or cognitive deficits, some researchers proposed that lowering endogenous tau is a beneficial treatment to protect neurons from Aβ toxicity [76]. However, our results imply that such tau-reducing therapy would result in failure to prevent Aβ-induced spine alteration.

Although AβOs generated by the Osaka mutation are mostly intracellular, picomolar amounts are released from cells (ELISA data; this study). We found that extracellular wild-type Aβ showed a trophic effect on synapses at physiological low concentrations; however, Osaka-mutant Aβ showed no trophic effect on spines. Recent evidence suggests that endogenous Aβ modulates synaptic plasticity [40, 41] and regulates neurotransmitter release probability [77]. These positive effects of Aβ are observed at picomolar concentrations, and higher, nanomolar concentrations lead to toxicity. In cultured hippocampal slices, we previously observed that relatively low concentrations of wild-type Aβ enhanced the levels of synaptophysin, whereas Osaka-mutant Aβ did not [42]. In the present study, unlike wild-type Aβ, Osaka-mutant Aβ did not show trophic effects on spines at the same low concentrations. The lack of such trophic actions may in part account for the synaptic alteration associated with APP_OSK_.

## Conclusion

In summary, we showed that iAβOs reduce mature dendritic spines and block axonal and dendritic transport in primary neurons. Both the spine alteration and BDNF transport blockade are tau-independent. iAβO-induced spine alterations may be caused by deregulating enzymes within dendritic spines and/or by reducing axonal and dendritic transport of organelles and proteins necessary for spine formation and maintenance. Furthermore, extracellular Osaka-mutant Aβ lacks the trophic effect on spines, likely inflicting a dual assault on spines in AD. This work is significant because iAβO generation precedes extracellular Aβ accumulation and these data will promote further investigation of iAβO toxicity with the hope to develop therapeutic compounds for prevention and treatment of AD.

## Abbreviations

AD: Alzheimer’s disease
Aβ: Amyloid β
APP: Amyloid precursor protein
eAβOs: Extracellular Aβ oligomers
iAβOs: Intracellular Aβ oligomers
BDNF: Brain-derived neurotrophic factor
LTP: Long-term potentiation
LTD: Long-term depression
TfR: Transferrin receptor
EGFP: Enhanced green fluorescent protein
mRFP: Monomeric red fluorescent protein
eBFP: Enhanced blue fluorescent protein
EYFP: Enhanced yellow fluorescent protein
HBSS: Hank’s balanced salt solution
Rac1: Ras-related C3 botulinum toxin substrate 1
FAT: Fast axonal transport
