# Erratum: Intracellular amyloid β oligomers impair organelle transport and induce dendritic spine loss in primary neurons

**DOI:** 10.1186/s40478-016-0273-z

**Published:** 2016-01-28

**Authors:** T Umeda, EM Ramser, M Yamashita, K Nakajima, H Mori, MA Silverman, T Tomiyama

**Affiliations:** Department of Neuroscience, Osaka City University Graduate School of Medicine, 1-4-3 Asahimachi, Abeno-ku, Osaka, 545-8585 Japan; Core Research for Evolutional Science and Technology, Japan Science and Technology Agency, Kawaguchi, Japan; Department of Biological Sciences, Simon Fraser University, Burnaby, BC V5A 1S6 Canada; Department of Immunology, Osaka City University Graduate School of Medicine, Osaka, Japan; Department of Clinical Neuroscience, Osaka City University Medical School, Osaka, Japan

## Erratum

The original version of this article unfortunately contained a mistake in the presentation of Fig. 1 in both the PDF and HTML versions of this manuscript [1]. In the right panel of the corrected Fig. 1d, the images of Mock cells, which were visualized with GFP and stained with Abeta oligomer-specific antibody 11A1, were replaced with those of APPWT cells, and instead the images of APPWT cells were replaced with those of Mock cells. These images had been incorrectly placed in the original Fig. 1. The correct version of Fig. 1 is presented below.

**Fig. 1:**
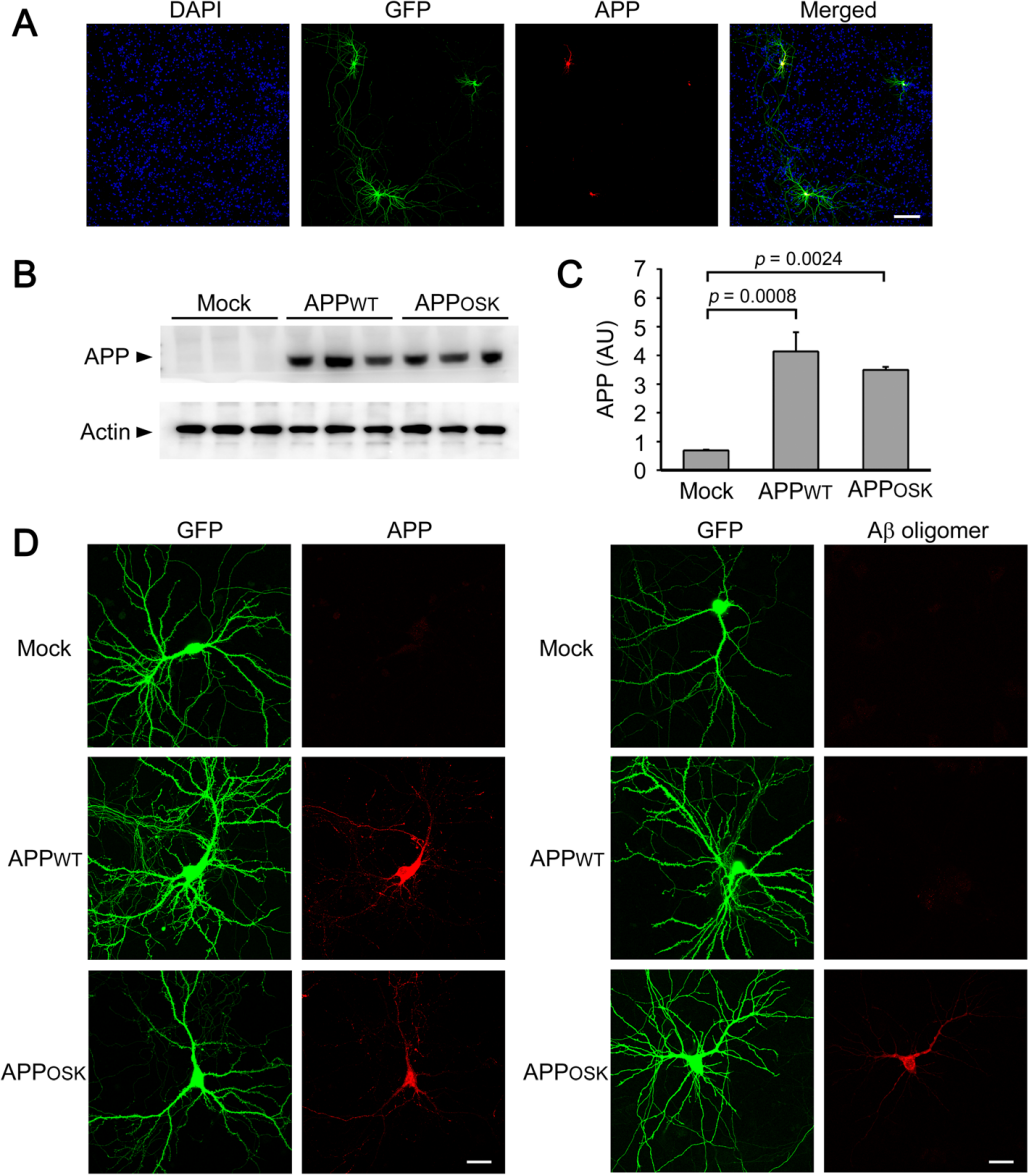
Intracellular amyloid β oligomers impair organelle transport and induce dendritic spine loss in primary neurons
